# Books and Magazines

**Published:** 1914-01

**Authors:** 


### Books and Magazines.

The Practical Medicine Series: Comprising ten volumes on the year's progress in Medicine and Surgery. Under the general editorial charge of Charles L. Mix, A. M., M. D., Professor of Physical Diagnosis in the Northwestern University Medical School. Vol. VI, General Medicine, edited by Frank Billings, M. S., M. D., head of the Medical Department and Dean of the faculty of Rush Medical College, Chicago, and J. H. Salisbury, A. M., M. D., Professor of Medicine, Chicago Clinical School. Series 1913. The Year Book Publishers, 327 S. LaSalle Street, Chicago.

The price of this volume is $1.50. Of the set of ten, ten dollars.

Anatomy and Dissector in Abstract, by Stewart L. McCurdy, A. M., M. D., Professor of Anatomy and Surgery (Dental Department) University of Pittsburg. Fourth edition. Medical Abstract Publishing Co., Pittsburg.

This little manual is what it purports to be, a kind of epitome of anatomy and a guide to dissection, intended for students and for reference by practitioners. No price given.

---

Pocket Cyclopedia of Medicine and Surgery. Gould & Pyle. Second edition, revised, enlarged and edited by R. J. E. Scott, M. A., B. C. L., M. D., New York. Philadelphia, P. Blakiston's Son & Co. Price $1.00.

This handy little volume is epitomised from Gould & Pyle's well known and popular Cyclopedia of Practical Medicine and Surgery, and will be found very useful for quick reference. It is arranged alphabetically and covers the entire field of practice.

---

Treatment of Internal Diseases for Physicians and Students: By Prof. Norbert Ortner, of the University of Vienna; edited, with additions, by Nathaniel Bowditch Potter, M. D., Assistant Professor of Clinical Medicine at Columbia University (College of Physicians and Surgeons), New York; visiting physician to the New York City Hospital and to the French Hospital. Translated by Frederick H. Bartlett, M. D., Instructor in Children's Diseases at Columbia University (College of Physicians and Surgeons), New York, and attending physician to the Babies Hospital. Second edition in English; revised and reset from the Fifth German Edition. J. B. Lippincott Company, New York and London, 1913. Cloth, 610 pages. Price $5.

This is a most admirable book. It is practical. It tells one what to do in every kind of internal disease and that is what one wants to know. There is no speculation, or "ifs and ands" about it. The author goes straight at the malady under consideration, tells you all about it, quotes our own Mayo and Dock and numerous other noted clinicians. Reading it, or reading up on any particular subject is almost as good as attending a clinic. I quote from the editor's preface:

"The scope of the book is indicated in its title. The author initiates the reader into as much of the pathological physiology of the diseases discussed, as bears upon their rational treatment." (Pathological *physiology* is good.) "He very properly emphasizes throughout the volume the importance of the mechanical, dietetic, climatic, hydrotherapeutic, and other extra-medicinal methods, with judicious reasons for those selected, and follows these with a discussion of the applicability of certain drugs, their respective advantages, disadvantages and limitations, adds useful prescriptions from his own experience and that of others, and leaves the reader better armed to meet etiological indications and the various contingencies which arise and require symptomatic treatment."

The subject is too large for a comprehensive review, but enough has been said to impress readers with my conception of the value of the book. Buy it. $5.00.

---

FIRST BOOK OF HEALTH, a text-book of personal hygiene for pupils in the lower grades. By Carl Hartman, B. A., M. A. Instructor in Zoology, University of Texas, and Lewis Bradley Bibb, B. A., M. D., Attending Physician, Austin Sanitarium. With 122 illustrations. Price not stated. World Book Company, Yonkers-on-Hudson, New York, 1913. And

THE HUMAN BODY AND ITS ENEMIES, A Text-book of physiology, hygiene and sanitation by the same authors and the same publishers. The mailing price of this book is 84 cents.

It is sufficient to say that these two little books have been examined by the Texas School Book Board and adopted for use in the Texas free schools.

---

PSYCHOPATHIA SEXUALIS with Especial Reference to the Antipathic Sexual Instinct; a Medico-Forensic Study. By Dr. R. von Krafft-Ebing, o. o. Prof. fur Psychiatrie und Nervenkrankheiten an der K. K. University Wien. Only authorized English adaptation of the 12th German Edition. By F. J. Rebman, with author's portrait as frontispiece. New York, Rebman Company, Publishers. Cloth $4.00.

For review see editorial pages.

---

PREVENTIVE MEDICINE AND HYGIENE: By Milton J. Rosenau, Professor of Preventive Medicine and Hygiene, Harvard. Formerly Director of the Hygienic Laboratory, U. S. P. H. Service. With chapters on Sewage and Garbage, by Geo. C. Whipple, Professor of Sanitary Engineering, Harvard. Vital Statistics, by Cressy L. Wilbur, Chief Statistician, Bureau of the Census, Department of Commerce and Labor. The Prevention of Mental Diseases, by Thomas W. Salmon, Director of Special Studies, National Committee for Mental Hygiene, etc. New York and London. D. Appleton & Co., 1913. Handsomely bound, 1034 pages. Price, $5.00.

This is indeed a great book—a monumental book. It contains about all that anyone will ever have occasion to know or want to know, on the subject of Preventive Medicine and Hygiene, and all that is incidental or contributory or collateral thereto, in the chapters on Sewage and Garbage, Vital Statistics, the Prevention of Mental Diseases, all written, and splendidly written by masters in their several special branches of learning. It would be weak to say the work has been excellently well done, and superfluous to say the publishers have done the mechanical part of the work in a manner well up to the Appleton standard. Now that sanitation is a world wide movement and prevention is taking the place of cure and drugs in the practice of medicine every active physician should hasten to buy this great book.

